# *Peromyscus* mice as a model for studying natural variation

**DOI:** 10.7554/eLife.06813

**Published:** 2015-06-17

**Authors:** Nicole L Bedford, Hopi E Hoekstra

**Affiliations:** Department of Organismic and Evolutionary Biology, Harvard University, Cambridge, United States; Department of Molecular and Cellular Biology, Harvard University, Cambridge, United States; Museum of Comparative Zoology, Harvard University, Cambridge, United States and Howard Hughes Medical Institute, Harvard University, Cambridge, United States; Department of Organismic and Evolutionary Biology, Harvard University, Cambridge, United States; Department of Molecular and Cellular Biology, Harvard University, Cambridge, United States; Museum of Comparative Zoology, Harvard University, Cambridge, United States and Howard Hughes Medical Institute, Harvard University, Cambridge, United States

**Keywords:** the natural history of model organisms, *Peromyscus*, evolution, ecology, natural history, adaptation, mouse

## Abstract

The deer mouse (genus *Peromyscus*) is the most abundant mammal in North America, and it occupies almost every type of terrestrial habitat. It is not surprising therefore that the natural history of *Peromyscus* is among the best studied of any small mammal. For decades, the deer mouse has contributed to our understanding of population genetics, disease ecology, longevity, endocrinology and behavior. Over a century's worth of detailed descriptive studies of *Peromyscus* in the wild, coupled with emerging genetic and genomic techniques, have now positioned these mice as model organisms for the study of natural variation and adaptation. Recent work, combining field observations and laboratory experiments, has lead to exciting advances in a number of fields—from evolution and genetics, to physiology and neurobiology.

**DOI:** http://dx.doi.org/10.7554/eLife.06813.001

## Introduction

*Peromyscus* is a genus of small North American rodents known colloquially as deer mice (Emmons, 1840). When the first *Peromyscus* specimens were shipped to European systematicists in the late 18th century, their resemblance to the local wood mouse prompted the designation *Mus sylvaticus* (Hooper, 1968). At the time, little was known of the diversity of rodents worldwide and most were assigned the generic term *Mus* (Linnaeus, 1758). The name *Peromyscus* (Gloger, 1841) was first employed, albeit narrowly, in the middle of the 19th century. *Quadrupeds of North America* (Audubon and Bachman, 1854) recognized only three species now known to belong to *Peromyscus*, and *Mammals of North America* (Baird, 1859) included a mere 12. But by the turn of the 20th century, *Peromyscus* included 143 forms, 42 of which represented monotypic or good biological species (Osgood, 1909). The genus saw several additional revisions throughout the 20th century as North American mammalogy matured and natural history collections expanded. Today 56 species are recognized, the most widespread and diverse being *Peromyscus maniculatus* (Musser and Carleton, 2005).

**Figure 1.**
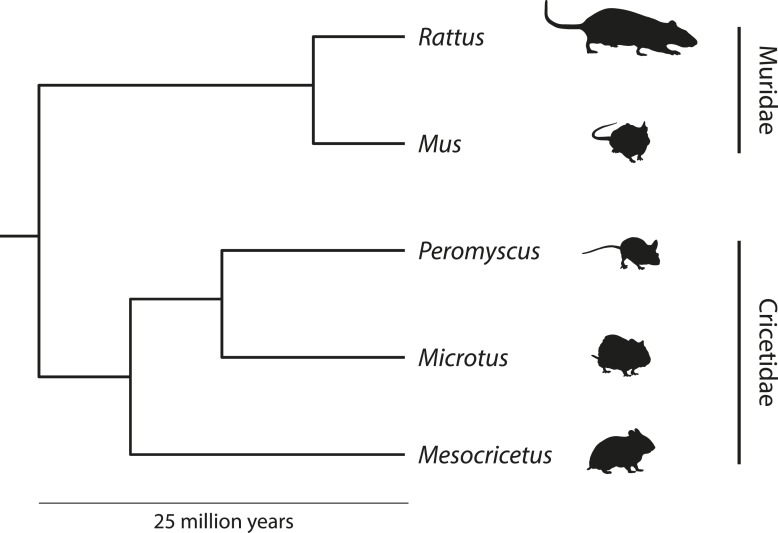
Simplified phylogeny depicting the relationships among muroid rodent model organisms. *Peromyscus* belong to the Cricetidae family, which includes voles (*Microtus*), hamsters (*Mesocricetus*), and New World rats and mice. Old World rats and mice belong to the Muridae family, which include the familiar laboratory rat (*Rattus norvegicus*) and mouse (*Mus musculus*). Muridae and Cricetidae diverged roughly 25 million years ago. Schematic based on based on phylogeny data from Steppan et al. (2004). Image credit, Nicole Bedford and Hopi Hoekstra. **DOI:** http://dx.doi.org/10.7554/eLife.06813.002

Thus, although not immediately appreciated, *Peromyscus* includes more species than any other North American mammalian genus and, apart from *Mus* and *Rattus*, more is known concerning its biology in the laboratory than any other group of small mammals (Figure 1; King, 1968; Kirkland and Layne, 1989). Several disciplines including ecology, evolution, physiology, reproductive biology and behavioral neuroscience have all employed *Peromyscus*, inspiring its label as ‘the *Drosophila* of North American mammalogy’ (Dewey and Dawson, 2001). Arguably, the emergence of *Peromyscus* as a model system was propelled by our cumulative knowledge of its fascinating and varied natural history.

## Distribution and habitat

‘*Within the range of one species* (maniculatus) *it is probable that a line, or several lines, could be drawn from Labrador to Alaska and thence to southern Mexico throughout which not a single square mile is not inhabited by some form of this species*’ (Osgood, 1909).

**Figure 2.**
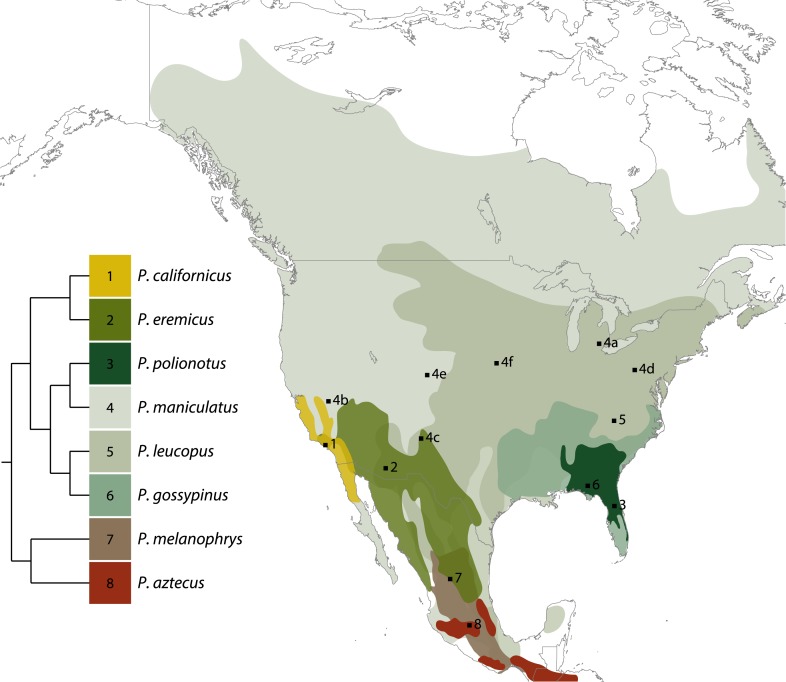
North American distributions of eight *Peromyscus* species currently maintained as outbred laboratory stocks (based on data from Hall, 1981). Some ranges are narrow and others are extensive, with many overlapping to a large extent. Simplified tree indicating phylogenetic relationships among taxa is shown; branch lengths are arbitrary (based on data from Bradley et al., 2007). The most widespread and ecologically diverse group is also the best represented in the laboratory: six *P. maniculatus* subspecies are maintained in laboratories across the United States. Collecting localities of colony founders are indicated by numbered squares (see also Table 2). Image credit, Nicole Bedford and Hopi Hoekstra. **DOI:** http://dx.doi.org/10.7554/eLife.06813.003

Wilfred H Osgood asserted that some form of *Peromyscus* had been trapped in nearly every patch of North America ever visited by a mammal collector. Members of the genus are distributed from the southern edge of the Canadian Arctic to the Colombian border of Panama (Figure 2). Various demographic and biogeographic factors (e.g., Pleistocene glacial and pluvial cycles, population expansions, mountain range elevations and sea-level changes) have influenced the diversity and distribution of deer mice (Sullivan et al., 1997; Riddle et al., 2000; Dragoo et al., 2006; Kalkvik et al., 2012; López-González et al., 2014). The result is a mosaic of widespread and restricted species ranges shaped by both dispersal and vicariance events. Our knowledge of the distributions, home ranges and habitat preferences of deer mice comes primarily from the trapping data and field notes of early natural historians (e.g., Sumner, 1917; Dice, 1931; Blair, 1940, 1951). Osgood's influential 1909 taxonomic revision was built on examinations of more than 27,000 specimens from diverse locales that were collected primarily by the US Biological Survey. Today, more than 120,000 *Peromyscus* specimens are accessioned in Natural History museums across North America and the United Kingdom (Table 1). These invaluable collections document more than a century of dynamic relationships between deer mice and their environment. For example, by comparing past and present-day collecting locales, shifts in the distributions of deer mice have been linked to climate change (Moritz et al., 2008; Yang et al., 2011; Rowe et al., 2014), and morphological analyses of these museum specimens reveal how deer mice respond to changing environments (Grieco and Rizk, 2010).

**Table 1.** Museums with the largest collections of *Peromyscus* specimens **DOI:** http://dx.doi.org/10.7554/eLife.06813.004

Collection	Location	No. specimens
Smithsonian National Museum of Natural History	Washington, DC	38,406
Museum of Vertebrate Zoology	Berkeley, CA	34,131
American Museum of Natural History	New York, NY	19,234
Field Museum	Chicago, IL	8939
Museum of Comparative Zoology	Cambridge, MA	7754
Canadian Museum of Nature	Ottawa, ON	6315
Academy of Natural Science	Philadelphia, PA	2425
Natural History Museum	London, UK	2238

**Figure 3.**
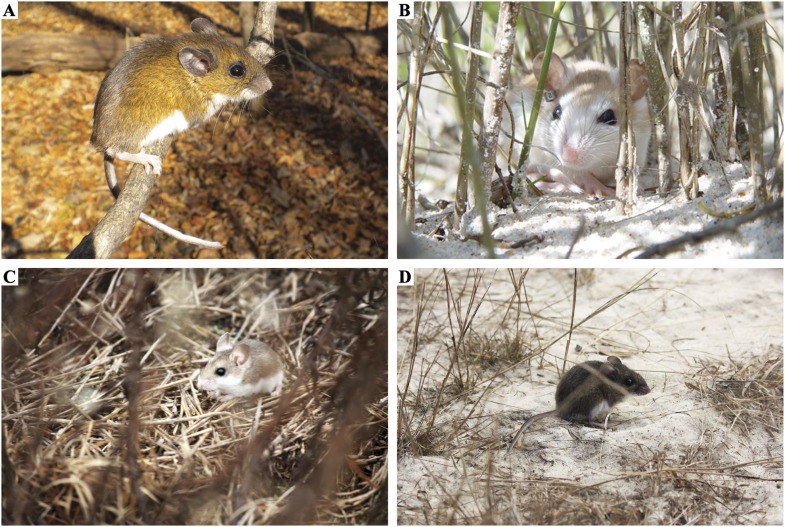
The ecology of *Peromyscus* varies considerably both within and among species. (**A**) The forest-dwelling deer mouse, *P. maniculatus nubiterrae*, perches high on a tree branch in Southwestern Pennsylvania. (**B**) The beach mouse, *P. polionotus phasma*, takes shelter among the dune grasses on Florida's Atlantic coast. (**C**) Its mainland counterpart, the oldfield mouse, *P. polionotus sumneri*, is typically found in fallow fields and is sympatric with the cotton mouse, *P. gossypinus* (**D**), which occupies adjacent stands of long leaf pine. Image credits: **A**, Evan P Kingsley; **B**, JB Miller; **C**, **D**, Nicole Bedford. **DOI:** http://dx.doi.org/10.7554/eLife.06813.005

Although not strictly commensal, deer mice (particularly in New England) do enter human households and partake of their larders. According to legend, Walt Disney drew inspiration for Mickey Mouse from the ‘tame field mice’ (most likely *Peromyscus leucopus*) that would wander into his old Kansas City animation studio (Updike, 1991). Nevertheless, *Peromyscus* are most commonly trapped in woodlands and brushlands and are also found in tropical and temperate rainforests, grasslands, savannas, swamps, deserts and alpine habitats (Figure 3; Baker, 1968). Local adaptation to these various environments has been the subject of much recent inquiry (e.g., Linnen et al., 2013; Natarajan et al., 2013; MacManes and Eisen, 2014), and the detailed cataloguing of phenotypic diversity by early naturalists inspired much of this work. However, we still require a more complete understanding of ecological diversity across the entire genus, as well as an enlightened view of phylogenetic relationships informed by whole-genome sequences (see Box 1).

10.7554/eLife.06813.006

Box 1. **Priorities for *Peromyscus* research**

**Discovering as yet untapped ecological diversity**

Much of our understanding of *Peromyscus* biology comes from studies of two ubiquitous species that have proven amenable to laboratory life—*P. maniculatus* and *P. leucopus*. However, most *Peromyscus* species remain comparatively understudied, particularly in Central America and Mexico where taxonomic diversity and endemism (i.e., where species are unique to a given geographic location) is greatest.

**Sequencing more *Peromyscus* genomes and revising their phylogeny**

A comprehensive phylogeny based on genome-wide DNA sequences would greatly facilitate the comparative approaches that are the unique advantage of the *Peromyscus* system. An annotated genome assembly is currently available for *P. maniculatus bairdii* (Pman_1.0, GenBank assembly accession GCA_000500345.1) and draft sequences are available for *P. californicus*, *P. leucopus* and *P. polionotus* (Baylor College of Medicine, www.hgsc.bcm.edu/peromyscus-genome-project). Several more *Peromyscus* genomes are being sequenced, but still more are needed.

**Identifying where *Peromyscus* can complement biomedical studies of other laboratory species**

The genetically diverse *Peromyscus* could be used more widely in biomedical research than previously thought. Indeed, certain aspects of human biology—including aging, epigenetics, retinal development and hematology—have been suitably modeled in *Peromyscus* (e.g., Ungvari et al., 2008; Shorter et al., 2012; Arbogast et al., 2013; Sun et al., 2014).

**DOI:** http://dx.doi.org/10.7554/eLife.06813.006

## Adaptation to mountains, cities and deserts

Among North American mammals, the deer mouse is unparalleled in its ability to colonize an impressive array of habitats. The remarkable elevational range of one subspecies (*P. m. sonoriensis*) stretches from below sea level in Death Valley to above 4300 meters in the adjacent White and Sierra Nevada mountain ranges (Hock, 1964). The ability of deer mice to colonize and thrive in low-oxygen environments is due, in part, to standing genetic variation in globin genes (Snyder, 1981; Natarajan et al., 2015). Storz and colleagues (2007, 2009) pinpointed several amino acid substitutions that confer high hemoglobin-O_2_ affinity and better aerobic performance at high altitudes. Functional analyses have since identified how precise mutations, and interactions among mutations, affect hemoglobin-O_2_ affinity, demonstrating that the adaptive value of a given biochemical substitution depends both on the local environment and the genetic background in which it arises (Natarajan et al., 2013).

The process of adapting to urban environments also leaves its mark on the genome (Pergams and Lacy, 2008; Munshi-South and Kharchenko, 2010; Munshi-South and Nagy, 2014). By comparing the brain, liver and gonad transcriptomes of urban and rural populations of *P. leucopus*, Harris *et al.* (2013) identified several genes associated with metabolism and immune function exhibiting signatures of selection in New York City's parklands. Similarly, MacManes and Eisen (2014) identified renal transcripts related to solute and water balance experiencing purifying selection in the desert-adapted species, *Peromyscus eremicus*. Further study of these candidate genes will determine their role in adaptation to new or extreme environments.

## Diet and predators

Generally deer mice are granivores, feeding primarily on seeds, but fruits, fungi, green vegetation and insects have been found among their stomach contents and in the nest cavities of their burrows (Gentry and Smith, 1968; Wolff, 1985). However, some species have evolved seasonally specialized diets. In the winter, *Peromyscus melanotis* prey almost exclusively on monarch butterflies that roost in Mexico's central highlands (Brower et al., 1985). Moreover, on a remote island in British Columbia, *Peromyscus keeni* feast on auklet eggs during the seabird breeding season (Drever et al., 2000). Deer mice are themselves common prey, contributing to the diets of many predators such as weasels, skunks, lynx, bobcats, foxes, coyotes, hawks and owls (Luttich et al., 1970; Bowen, 1981; Montgomery, 1989; Van Zant and Wooten, 2003). Indeed, avian predation imposes strong selective pressure for cryptic coloration in *Peromyscus*—a classic example of local adaptation (Vignieri et al., 2010; Linnen et al., 2013).

## Parasites and disease

The diversity of parasites is documented for only a few *Peromyscus* species, and very little is known of the ecological factors that influence infection dynamics. Common internal parasites include pentastomid larvae, cestode tapeworms, nematodes and trematodes (Whitaker, 1968; Pedersen and Antonovics, 2013). External parasites include lice, mites, fleas and ticks (Whitaker, 1968), the latter two being vectors of plague and Lyme disease, respectively (Allred, 1952; Burgdorfer et al., 1982; Gage and Kosoy, 2005).

As a natural reservoir for *Borrelia burgdorferi*—the bacterial agent of Lyme disease—*Peromyscus* is the subject of much research on the pathogenesis and transmission of the disease (Bunikis et al., 2004; Ramamoorthi et al., 2005; Schwanz et al., 2011; Baum et al., 2012). *Peromyscus* also features in ecological modeling efforts to determine how the diversity of the tick host community impacts disease risk (LoGiudice et al., 2003, 2008). One hypothesis for the alarming recent expansion of Lyme disease is that habitat fragmentation associated with human development favors deer mouse populations at the expense of other tick hosts (e.g., squirrels and shrews) that are poor reservoirs for the disease (LoGiudice et al., 2003; Schwanz et al., 2011). *Peromyscus* is also a notorious carrier of the Sin Nombre hantavirus, responsible for the deaths of 12 people in the Four Corners area of the southwestern United States in 1993.

## Longevity

Mortality in natural populations is incredibly high and driven by a combination of factors including limited food supply, competition for territories and predation (Bendell, 1959). As such, most *Peromyscus* are thought to live less than a year in the wild (Terman, 1968). However, early investigators noted substantially longer natural lifespans in their laboratory colonies (Sumner, 1922; Dice, 1933). With a twofold difference in life expectancy, Sacher and Hart (1978) proposed *P. leucopus* and *Mus musculus* as a longevity contrast pair. *P. leucopus*—which lives up to 8 years and may remain fertile for 5—produces fewer reactive oxygen species, exhibits enhanced antioxidant enzyme activity and less oxidative damage to lipids relative to the short-lived (~3.5 years) laboratory mouse (Sohal et al., 1993; Shi et al., 2013). Measuring the biochemical correlates of longevity in *Peromyscus* has been integral to providing support for the oxidative stress theory of aging (Ungvari et al., 2008).

## Life history

The timing of life history events in *Peromyscus*—well documented from field and laboratory studies alike—is highly variable both within and among species. Yet studies contrasting the reproductive and developmental patterns of wild and domesticated deer mice have found few significant differences (Millar, 1989; Botten et al., 2000). Here, we highlight life history traits in *P. maniculatus*, the most commonly used laboratory species. Gestation ranges from 21 to 27 days (average 23.6) and average litter size is 4.6 pups (Millar, 1989). Juveniles first leave the nest between 14 and 16 days of age (Vestal et al., 1980) and become independent of their mother between 18 and 25 days (Millar, 1989). Captive females give birth to their first litter, on average, at 84 days (Haigh, 1983), but males are capable of siring offspring several weeks earlier.

The actual timing of sexual maturation in the wild, however, is often dictated by population density, food availability and season. In response to short day length, many species exhibit seasonal gonadal regression (Trainor et al., 2006), increased aggression (Trainor et al., 2007), impaired spatial memory (Workman et al., 2009) and enhanced immune function (Prendergast and Nelson, 2001). As such, *Peromyscus* has emerged as a model system for the study of photoperiodism (i.e., the ability to seasonally modulate energetic demands by tracking day length changes). Such studies have been particularly fruitful for understanding the mechanistic basis of gene by environment interactions. For example, day length can reverse the behavioral action of the hormone estradiol by determining which estrogen receptor pathway is expressed and consequently activated (Trainor et al., 2007). While life history traits are strongly affected by environmental cues, substantial genetic variation in the neuroendocrine pathways that control reproductive timing also exists, as demonstrated by selection line experiments with photoperiod responsive and nonresponsive *P. leucopus* (Heideman et al., 1999; Heideman and Pittman, 2009).

## Mating system and parental care

While the majority of *Peromyscus* species are promiscuous, monogamy has independently evolved at least twice in the genus (Turner et al., 2010). Both *Peromyscus californicus* (Gubernick and Alberts, 1987; Ribble, 1991) and *Peromyscus polionotus* (Smith, 1966; Foltz, 1981) are socially and genetically monogamous, and both males and females contribute to the care of offspring. *P. californicus*, in particular, has become an important neurobiological model for the study of male parental care (Bester-Meredith et al., 1999; Trainor et al., 2003; Lee and Brown, 2007; de Jong et al., 2009, 2010). As a complement, the ability of monogamous *P. polionotus* to hybridize with promiscuous *P. maniculatus* allows geneticists to identify the genetic basis of alternate mating systems and their associated phenotypes, from genomic imprinting (Vrana et al., 2000) to parental investment and reproductive traits (e.g., Fisher and Hoekstra, 2010).

Rosenfeld (2015) argues that parental and social behaviors are particularly vulnerable to endocrine disruption, as these traits are dependent upon the organizational and activational effects of androgens and estrogens. Mating system variation between closely related species of deer mice provides an opportunity to test this hypothesis. *P. maniculatus* males exposed to the endocrine disrupting compound bisphenol A (BPA) during development displayed reduced spatial learning and exploratory behavior—traits known to be associated with male–male competition for mates (Galea et al., 1996; Jašarević et al., 2011). However, these behaviors—which are not subject to sexual selection in females—were unaffected in BPA-exposed females. By contrast, sexual selection favors the evolution of mate guarding and territorial behavior in monogamous males, and it is these traits (rather than spatial learning or exploratory behavior) that are compromised by endocrine disruption in *P. californicus* (Williams et al., 2013).

## Home building

Behavioral genetics studies have historically been restricted to a handful of genetic model organisms that display behaviors of unclear ecological relevance (Fitzpatrick et al., 2005). Sufficient resources are now available—from a medium-density genetic linkage map (Kenney-Hunt et al., 2014) to draft genome sequences (Baylor College of Medicine, *Peromyscus* Genome Project)—that we can attribute natural variation in *Peromyscus* behavior to specific genetic variants. For instance, *P. maniculatus* and *P. polionotus* display considerable differences in stereotyped burrowing behavior. *P. maniculatus* digs short, simple burrows in contrast to the long, complex burrows constructed by *P. polionotus* that consist of an entrance tunnel, nest chamber and escape tunnel (Dawson et al., 1988; Weber et al., 2013). Remarkably, mice raised in the laboratory for several generations recapitulate the species-specific burrow architectures observed in nature (Video 1). Furthermore, the complex burrows of *P. polionotus* are derived (Weber and Hoekstra, 2009) and likely evolved through changes at only a handful of genetic loci, each affecting distinct behavioral modules (i.e., entrance tunnel length and escape tunnel presence; Weber et al., 2013). Next steps include isolating genetic variants, understanding their effects on the neural circuitry underlying burrowing behavior and quantifying the adaptive value of burrowing in the wild.

**Video 1.** Innate burrowing behavior in *Peromyscus* can be directly observed in a laboratory setting. Here, *P. polionotus* is busy constructing the long entrance tunnel of its complex burrow. Video credit, Nicole Bedford and Hopi Hoekstra. **DOI:** http://dx.doi.org/10.7554/eLife.06813.007

## Pigmentation

**Figure 4.**
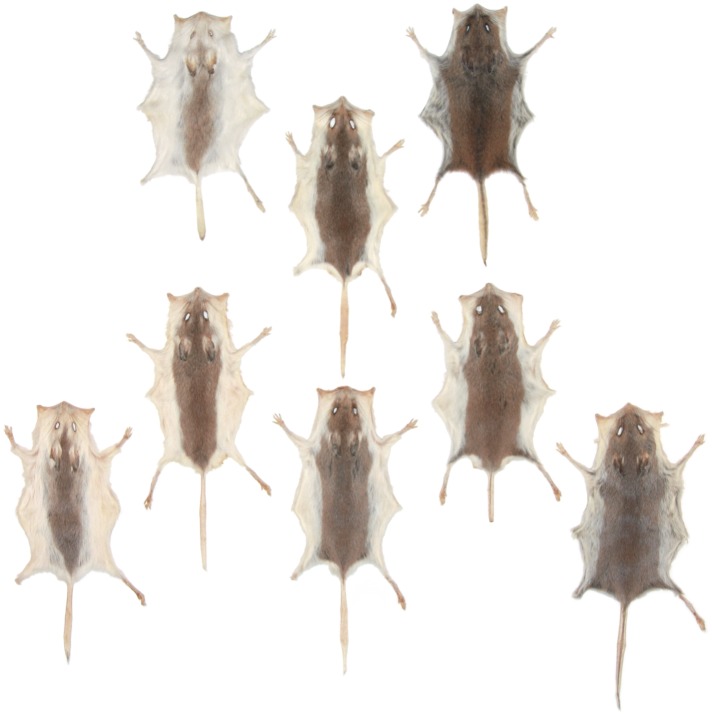
Genetic crosses between the pale beach mouse *P. polionotus leucocephalus* (top row left) and the darker mainland mouse *P. p. polionotus* (top row right) result in first-generation F_1_ hybrids, all with intermediate coloration (second row). Intercrosses between F_1_ hybrids produce a variable F_2_ generation, showing a continuous distribution of pigmentation phenotypes ranging from light to dark (third and fourth rows; Steiner et al., 2007). This segregation pattern—initially described by Francis Sumner—is among the earliest empirical evidence that several discrete loci may collectively contribute to a quantitative trait (Dobzhansky, 1937; see also Box 1). Image credit, Nicole Bedford and Hopi Hoekstra. **DOI:** http://dx.doi.org/10.7554/eLife.06813.008

Among the several cases of adaptive phenotypic variation in *Peromyscus*, perhaps the most obvious is coat coloration. Recent advances have identified not only the genes, but also the specific mutations, leading to local variation in coat color. Beach mice (*P. polionotus leucocephalus*) living on the coastal sand dunes and barrier islands of Florida are considerably paler than their inland counterparts (*P. p. subgriseus*) that inhabit dark, loamy soils (Figure 4; Howell, 1920; Sumner, 1929). For beach mice on Florida's Gulf Coast, light coloration is due, in part, to a fixed single nucleotide polymorphism (SNP) in the melanocortin-1 receptor (*Mc1r*) coding region (Hoekstra et al., 2006). However, this *Mc1r* allele does not contribute to light pelage in Florida's Atlantic coast mice, suggesting that the two populations converged on light coloration independently (Steiner et al., 2007).

Similarly, background matching in *P. maniculatus* of the Nebraska Sand Hills affords a strong selective advantage against avian predators (Linnen et al., 2013). Yet, cryptic coloration is a complex phenotype composed of multiple component traits (i.e., tail stripe, dorsal-ventral boundary, ventral color, dorsal brightness and hue). Linnen and colleagues (2013) identified multiple distinct mutations within the *Agouti* locus, each associated with a different color trait that independently affected fitness. Thus, parallel studies of *Peromyscus* pigmentation nicely illustrate the marriage between classical natural history studies and modern molecular techniques, thereby providing new insights into the molecular basis of adaptation.

## *Peromyscus* in the laboratory

Francis Sumner, considered the grandfather of *Peromyscus* biology (see Box 2), first demonstrated the feasibility of the deer mouse as a laboratory organism in the 1910s and 20s. He famously built the first *Peromyscus* ‘mouse house’ in what is now referred to as Sumner Canyon at the Scripps Institution in La Jolla, California. When his *Peromyscus* work at Scripps was discontinued, Sumner bequeathed his stocks to Lee R Dice at the University of Michigan who honed the methods for generating and maintaining *Peromyscus* colonies in the 1930s and 40s. During this time, Dice began to catalogue single factor genetic mutations in his stocks (e.g., *gray*, *dilute*, *epilepsy*). These mice served as the founding strains for the Peromyscus Genetic Stock Center (PGSC) established in 1985 by Wallace Dawson at the University of South Carolina, which currently maintains wild-derived stocks of six species, as well as 13 coat-color mutants and four additional mutants on *P. maniculatus* genetic backgrounds. Additional wild-derived stocks are kept in individual laboratories (Table 2) and still more mutants have been cryopreserved. The PGSC also maintains an extensive online reference library (http://stkctr.biol.sc.edu) with more than 3000 citations.

**Table 2.** Current laboratory colonies of *Peromyscus* **DOI:** http://dx.doi.org/10.7554/eLife.06813.009

	Species	Year	Source population	Location
1	*P. californicus insignis*	1979–1987	Santa Monica Mts., CA	PGSC
2	*P. eremicus* sp.	1993	Tucson, AZ	PGSC
3	*P. polionotus subgriseus*	1952	Ocala National Forest, FL	PGSC
4a	*P. maniculatus bairdii*	1946–1948	Ann Arbor, MI	PGSC
4b	*P. m. sonoriensis*	1995	White Mtn. Research Station, CA	PGSC
4c	*P. m. rufinus*	1998	Manzano Mtn., NM	UNM
4d	*P. m. nubiterrae*	2010	Powder Mill Nature Reserve, PA	HU
4e	*P. m. rufinus*	2014	Mt. Evans, CO	UIUC
4f	*P. m. nebrascensis*	2014	Lincoln, NE	UIUC
5	*P. leucopus* sp.	1982–1985	Linville, NC	PGSC
6	*P. gossypinus gossypinus*	2009	Jackson County, FL	HU
7	*P. melanophrys xenerus*	1970–1978	Zacatecas, Mexico	UIUC
8	*P. aztecus hylocetes*	1986	Sierra Chincua, Mexico	UIUC

The year and population from which the founders were collected are noted. Numbers refer to collecting localities shown in Figure 2. PGSC: Peromyscus Genetic Stock Center; UNM: University of New Mexico; HU: Harvard University; UIUC: University of Illinois at Urbana-Champaign.

10.7554/eLife.06813.010

Box 2. ***Peromyscus* and the history of evolutionary thought**

The work of early *Peromyscus* biologists (particularly Francis B Sumner) informed influential thinkers in population genetics and evolutionary biology, such as Sewall Wright, Theodosius Dobzhansky and JBS Haldane. Since most early 20th century geneticists came from experimentalist backgrounds, many turned to naturalists for data from wild populations (Provine, 1986). At the time, Sumner's work on geographic variation in *Peromyscus* represented one of the few major studies of evolution in natural populations. As such, Wright closely followed Sumner's analysis of phenotypic intergradation between geographically contiguous *P. maniculatus* subspecies in California (Sumner, 1918). Wright concluded that the observed quantitative differences in coat color were determined by the accumulation of several discrete (i.e., Mendelian) factors (Wright, 1932). The question of whether continuous (or quantitative) traits are subject to the same rules of inheritance as discrete characters was central to the Modern Evolutionary Synthesis.

Between 1914 and 1930, Sumner carefully measured several quantitative traits—most notably coat color—that varied among geographically distinct subspecies of *Peromyscus*, which he then crossed in the laboratory (Figure 4; Sumner, 1930). Dobzhansky (1937) highlighted these data as empirical support for the multiple gene hypothesis for the inheritance of quantitative traits. Later, Haldane (1948) applied a theoretical model to the gradient of increasing pigmentation observed in *P. polionotus* populations from coastal to inland Florida (Sumner, 1929). From these data, he estimated the local strength of selection acting on a putative pigmentation locus in the wild—the dominant white-cheek character (Wc) identified by Blair (1944).

*Peromyscus* also featured in Dobzhansky's studies of reproductive isolation. Certain *P. maniculatus* subspecies with overlapping geographic distributions are nevertheless separated by habitat, often with one subspecies inhabiting prairie, open fields or sandy lake beaches, and the other being exclusively forest-dwelling (Dice, 1931). These sub-specific forms readily produce viable and fertile offspring in the laboratory yet remain reproductively isolated in the wild—a prime example of ecological isolation (Dobzhansky, 1937). *Peromyscus* has thus been a cornerstone of evolutionary biology for nearly a century. These and other studies drew the attention of biologists in many fields, launching the many, varied *Peromyscus* research programs we see today.

**DOI:** http://dx.doi.org/10.7554/eLife.06813.010

While the genetic causes and phenotypic consequences differ among strains, *Peromyscus* colonies are invariably susceptible to inbreeding depression, which necessitates their maintenance as relatively outbred stocks (Lacy et al., 1996; Joyner et al., 1998). Thus, although the deer mouse is amenable to laboratory life, its biology has not been purposely altered by generations of inbreeding or artificial selection. Life history traits and even behaviors such as burrow construction or ultrasonic vocalization are generally preserved in laboratory strains (Dawson et al., 1988; Millar, 1989; Kalcounis-Rueppell et al., 2010). Thus, the traits we scrutinize in the laboratory (e.g., aerobic performance, photoperiodism, mating and parental behavior) are arguably faithful representations of phenotypes in nature. The ability to study genetically diverse, wild-derived mice under controlled laboratory conditions has opened up several constructive research programs centered on understanding the phenotypic consequences of natural genetic variation.

## Conclusions

The tradition of dissecting the genetic basis of ecologically relevant traits in the laboratory began in the early 20th century; in *Peromyscus*, this effort was lead by Francis Sumner and continues today. In an era of high-throughput sequencing and expanding transgenic technologies, our concept of the genetic model organism is rapidly changing. We can now widen our focus to include the diverse and naturally evolving species that may further our understanding of life outside the laboratory. The emergence of *Peromyscus* as a model system has been largely driven by the wealth of natural history information available for the genus. Indeed, deer mice form the foundation of much of our understanding of the biology of small mammals. The multitude of ecological conditions to which deer mice have adapted has contributed to an impressive array of biological diversity within a single, ubiquitous genus. While this radiation is fascinating in its own right, *Peromyscus* is arguably foremost among nascent model systems that may aptly model the genetic complexity of the human condition, which too has long been shaped by natural selection in the wild. We hope that the continued development—primarily through the growth of genetic and genomic resources—of this model system will galvanize research in all corners of biology.
